# MRI-Based Model for Personalizing Neoadjuvant Treatment in Breast Cancer

**DOI:** 10.3390/tomography11030026

**Published:** 2025-02-27

**Authors:** Wen Li, Natsuko Onishi, Jessica E. Gibbs, Lisa J. Wilmes, Nu N. Le, Pouya Metanat, Elissa R. Price, Bonnie N. Joe, John Kornak, Christina Yau, Denise M. Wolf, Mark Jesus M. Magbanua, Barbara LeStage, Laura J. van ’t Veer, Angela M. DeMichele, Laura J. Esserman, Nola M. Hylton

**Affiliations:** 1 Department of Radiology and Biomedical Imaging, University of California, San Francisco, CA 94158, USA; natsuko.onishi@ucsf.edu (N.O.); jessica.gibbs@ucsf.edu (J.E.G.); lisa.wilmes@ucsf.edu (L.J.W.); nu.le@ucsf.edu (N.N.L.); pouya.metanat@ucsf.edu (P.M.); elissa.price@ucsf.edu (E.R.P.); bonnie.joe@ucsf.edu (B.N.J.); nola.hylton@ucsf.edu (N.M.H.); 2 Department of Epidemiology and Biostatistics, University of California, San Francisco, CA 94158, USA; john.kornak@ucsf.edu; 3 Department of Surgery, University of California, San Francisco, CA 94158, USA; cyau@buckinstitute.org (C.Y.); laura.esserman@ucsf.edu (L.J.E.); 4 Department of Laboratory Medicine, University of California, San Francisco, CA 94158, USA; denise.wolf@ucsf.edu (D.M.W.); mark.magbanua@ucsf.edu (M.J.M.M.); laura.vantveer@ucsf.edu (L.J.v.’t.V.); 5 I-SPY 2 Advocacy Group, San Francisco, CA 94158, USA; blestage51@gmail.com; 6 Department of Medicine, University of Pennsylvania, Philadelphia, PA 19104, USA; angela.demichele@pennmedicine.upenn.edu

**Keywords:** neoadjuvant chemotherapy, breast cancer, dynamic contrast-enhanced MRI, therapy decision, functional tumor volume, pathologic complete response

## Abstract

Background: Functional tumor volume (FTV), measured from dynamic contrast-enhanced MRI, is an imaging biomarker that can predict treatment response in breast cancer patients undergoing neoadjuvant chemotherapy (NAC). The FTV-based predictive model, combined with core biopsy, informed treatment decisions of recommending patients with excellent responses to proceed to surgery early in a large NAC clinical trial. Methods: In this retrospective study, we constructed models using FTV measurements. We analyzed performance tradeoffs when a probability threshold was used to identify excellent responders through the prediction of pathology complete response (pCR). Individual models were developed within cohorts defined by the hormone receptor and human epidermal growth factor receptor 2 (HR/HER2) subtype. Results: A total of 814 patients enrolled in the I-SPY 2 trial between 2010 and 2016 were included with a mean age of 49 years (range: 24 to 77). Among these patients, 289 (36%) achieved pCR. The area under the ROC curve (AUC) ranged from 0.68 to 0.74 for individual HR/HER2 subtypes. When probability thresholds were chosen based on minimum positive predictive value (PPV) levels of 50%, 70%, and 90%, the PPV-sensitivity tradeoff varied among subtypes. The highest sensitivities (100%, 87%, 45%) were found in the HR−/HER2+ sub-cohort for probability thresholds of 0, 0.62, and 0.72; followed by the triple-negative sub-cohort (98%, 52%, 4%) at thresholds of 0.13, 0.58, and 0.67; and HR+/HER2+ (78%, 16%, 8%) at thresholds of 0.34, 0.57, and 0.60. The lowest sensitivities (20%, 0%, 0%) occurred in the HR+/HER2− sub-cohort. Conclusions: Predictive models developed using imaging biomarkers, alongside clinically validated probability thresholds, can be incorporated into decision-making for precision oncology.

## 1. Introduction

For women diagnosed with breast cancer and treated with neoadjuvant chemotherapy (NAC), achieving pathologic complete response (pCR) after NAC predicts favorable long-term survival [1,2]. NAC enables real-time assessment of tumor treatment response and offers opportunities for treatment optimization to maximize an individual patient’s chance of achieving pCR. Patients with excellent responses after initial therapy might be considered for early surgery, avoiding additional treatment and minimizing unnecessary toxicity.

Dynamic contrast-enhanced (DCE) MRI is one of the most accurate tools for assessing breast cancer response to NAC [3,4]. Functional tumor volume (FTV), derived from DCE-MRI, is a quantitative imaging marker established by the American College of Radiology Imaging Network (ACRIN) 6657 multicenter clinical trial as a non-invasive measure of breast tumor burden. Change in FTV is superior to clinical assessment and MR longest diameter for predicting pCR [5]. Based on these findings, FTV is used to non-invasively assess breast cancer response to neoadjuvant therapies in the ongoing I-SPY 2 TRIAL (Investigation of Serial Studies to Predict Your Therapeutic Response with Imaging And moLecular analysis 2, *ClinicalTrials.gov* ID: NCT01042379), a multicenter, phase 2 neoadjuvant platform trial for high-risk, early-stage breast cancer.

Breast cancer is heterogeneous clinically and biologically [6,7]. Clinically, breast cancer is often categorized into four subtypes defined by hormone (estrogen or progesterone) receptor (HR, positive/negative) and human epidermal growth factor receptor 2 (HER2, positive/negative) and treated accordingly. The predictive performance of imaging biomarkers varies by breast cancer subtype [8,9,10]. However, the clinical implementation of this variability is still not clear.

In general, imaging-based models can be used to predict treatment responses regarding pathologic outcomes such as pCR or event-free survival (EFS). However, changing a treatment by recommending that a patient proceed to surgery early needs patient-level performance evaluation with predictive probability threshold analysis. This study aimed to investigate the optimal performance parameters of predicting pCR using FTV-based models and to demonstrate how the model can be applied to make recommendations for patients to proceed to surgery early.

## 2. Materials and Methods

This retrospective study followed the Health Insurance Portability and Accountability Act (HIPAA) policy. All patients provided written informed consent prior to screening, and a second consent form after randomization to treatment and before treatment was initiated. All I-SPY 2 sites received approvals from the institutional review board of each site before recruiting patients.

### 2.1. Study Cohort

Adults aged 18 years or older and diagnosed with stage II or III breast cancer and a tumor size of 2.5 cm or larger by clinical examination or 2.0 cm or larger by imaging are eligible for I-SPY 2. To be eligible for enrollment, patients with HR+/HER2− breast cancer also need to be at high risk for recurrence based on the MammaPrint (Agendia, Irvine, CA, USA) genomic test. This study cohort was from the publicly available dataset of 990 patients enrolled in the I-SPY 2 trial from May 2010 to November 2016 [11,12]. Patients were randomized to the control arm consisting of weekly paclitaxel alone (plus trastuzumab [Herceptin] for HER2+ patients) or in combination with one of nine experimental agents, followed by doxorubicin (Adriamycin) and cyclophosphamide (Cytoxan) (AC). Patients were included if they met all of the following criteria: (1) completed treatment and surgery; (2) had pathological assessment at surgery (pCR or non-pCR); (3) had HR and HER status assessed before the start of the treatment; and (4) had FTV measurements from all three MRI examinations (T0: pretreatment, T1: early treatment, T2: inter-regimen). Causes for missing MRI examinations included protocol violation, incomplete data, severe motion or other artifacts, insufficient fat suppression, and low signal-to-noise [SNR] ratio.

### 2.2. Image Acquisition

DCE-MRI was performed by acquiring a series of three-dimensional fat-suppressed T1-weighted images bilaterally. See Table S1 for the detailed I-SPY 2 image acquisition protocol, which was standardized based on clinical breast MR imaging, our experience in the I-SPY 1 and 2 trials, and in compliance with the American College of Radiology (ACR) guidance [13]. To summarize, the imaging parameters were TR = 4–10 msecs, minimum TE, flip angle = 10–20°, in-plane resolution ≤ 1.4 mm, slice thickness ≤ 2.5 mm, temporal resolution = 80–100 s, field of view (FOV) = 260–360 mm to achieve full bilateral coverage, acquisition matrix = 384–512, and axial orientation. A gadolinium-based contrast agent was administrated intravenously at a dose of 0.1 mmol/kg using an injection rate of 2 mL/s, followed by a 20 mL saline flush. The pre-contrast and multiple post-contrast acquisitions were repeated using identical sequence parameters, and post-contrast imaging continued for at least 8 min after the contrast agent injection. The same image acquisition protocol, magnet configuration (vendor, field strength, breast coil), and contrast agent brand had to be used to acquire all exams for the same patient. The technologist at the I-SPY Imaging Core Lab assessed the image acceptability for processing of FTV calculations based on several quality factors (success of contrast injection, absence of participant motion, fat suppression, or other artifacts).

### 2.3. FTV Measurement

FTV (unit: cubic centimeter, cc) was calculated semiautomatically from DCE-MRI using in-house software tools developed by IDL (Interactive Data Language, version 8.4; NV5 Geospatial Software, Inc. Broomfield, Colorado). A three-dimensional volume-of-interest (VOI) was delineated manually on the subtracted image by the site radiologist or trained imaging coordinator. FTV was calculated as the sum of volumes of voxels within the VOI and with a percentage enhancement (PE) above 70% and a signal enhancement ratio (SER) above 0, where $PE=\frac{S_{1}-S_{0}}{S_{0}}\times 100\%$ and $SER=\frac{S_{1}-S_{0}}{{S_{2}-S}_{0}}$ (S_0_, S_1_, and S_2_ were signal intensities at pre-contrast, early post-contrast [approximately two minutes and 30 s after contrast injection], and late post-contrast [approximately seven minutes and 30 s after contrast injection]) [14]. The empirical PE and SER thresholds were 70% and 0 by default but can be adjusted to optimize the FTV segmentation visually at the baseline. An example of FTV measurement is shown in Figure S1. The exact VOI dimensions and enhancement thresholds were applied to all subsequent DCE-MRIs for the same patient. This study analyzed baseline FTV and percent changes in FTV at T1 and T2.

### 2.4. Receptor Status and Pathologic Outcome

Estrogen receptor (ER), progesterone receptor (PR), and HER2 status (positive or negative) were assessed at baseline. The entire study cohort was stratified based on HR and HER2 status: HR-positive (ER or PR positive, HR+) and HER2-negative (HER2−), HR+ and HER2+, HR− and HER2+, and HR− and HER2− (triple negative). Pathologic outcomes were assessed after the completion of NAC at the time of surgery. Residual cancer burden (RCB) was determined through pathological assessment, and pCR was defined as an RCB-0 [15]. This study used binary outcome (pCR versus non-pCR) to develop and evaluate predictive models.

### 2.5. Statistical Analysis

The two-sided *t*-test was used to compare continuous variables of patient characteristics, i.e., age. Fisher’s exact test was used for categorical variables, i.e., HR/HER2 subtype and menopausal status. Fisher’s exact test was justified when analyzing categorical variables with small sample sizes [16].

Predictive logistic regression models were developed to predict pCR (versus non-pCR). Subtype-specific models were built by selecting FTV variables in the logistic regression to achieve the highest area under the receiver operating characteristic curve for each subtype. See Table S2 and Figure S2 for the details of predictive models. To demonstrate the utilization of MRI predictive models in clinical decision-making, probability threshold analysis for achieving pCR was conducted for each model. To identify excellent responders, sensitivity (the proportion of true pCRs meeting the subtype-specific probability threshold) and PPV (the proportion of patients meeting the subtype-specific probability threshold who were true pCRs) were the two performance parameters of interest. Between PPV and sensitivity, the former was more important in making recommendations for proceeding to surgery early because we expect to keep a minimal mistake of recommending a non-pCR (false positive) to skip the planned treatment, which the patient may actually benefit, and to proceed to surgery early. Therefore, probability thresholds were chosen by setting minimum PPV levels, which were set at values of 50%, 70%, and 90%, to demonstrate the tradeoff between PPV and sensitivity. All statistical analyses were conducted in R version 4.2.2 (R Foundation for Statistical Computing, Vienna, Austria). Results were considered statistically significant at *p* < 0.05.

## 3. Results

### 3.1. Study Cohort

**Figure 1:**
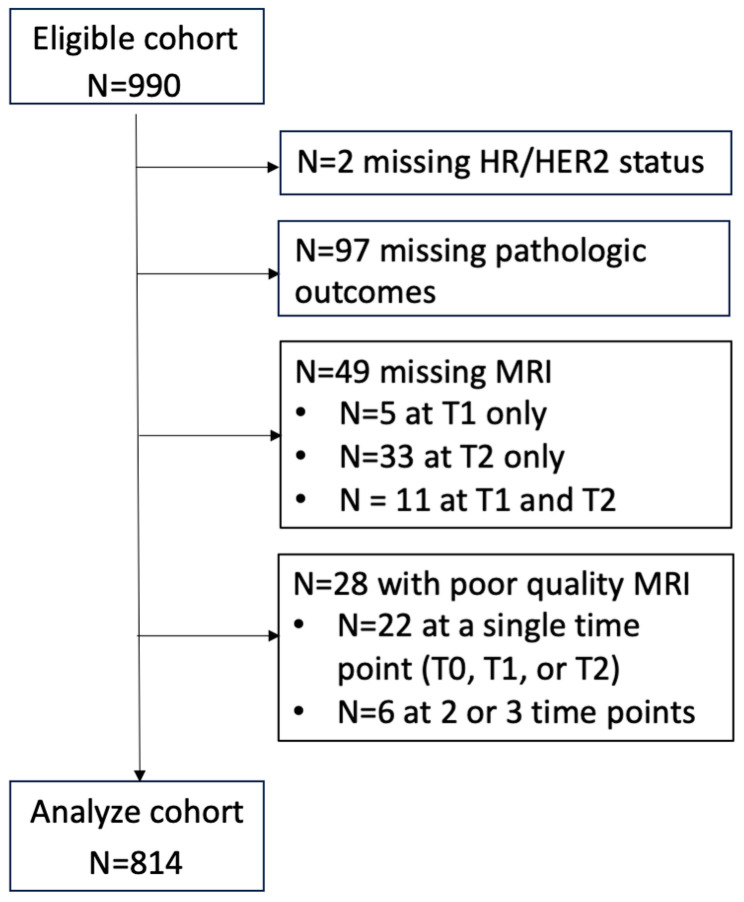
Flowchart of data exclusion and inclusion. Patients with missing data and with poor quality MRIs were excluded from the analysis. HR: hormone receptor. HER2: human epidermal growth factor receptor 2. T0: pretreatment time point. T1: early treatment time point (approximately 3 weeks after the start of treatment. T2: inter-regimen (approximately 12 weeks after the start of treatment).

Of the study cohort composed of 990 patients, clinical data and imaging data of 814 patients were included in the analysis. Patients with missing data were excluded from the analysis. Ninety-nine patients were excluded due to missing pCR outcomes or HR/HER2 status. An additional 49 patients were excluded because of missing MRI data, and 28 patients were excluded because of poor MR image quality (9 for motion or other artifacts, 1 for insufficient fat suppression, 3 for low SNR, and 15 for protocol violation). See Figure 1 for data exclusion details. Patient characteristics (age, HR/HER2 subtype, menopausal status, and pathologic outcome) are listed in Table 1 for the eligible cohort (n = 990) and analysis cohort (n = 814). The mean age was 49 years, and the standard deviation was 11 years for both cohorts. HR/HER2 subtype distributions and menopausal status were similar between the cohorts. However, the pCR and non-pCR rates were higher in the analysis cohort compared to the eligible cohort because the latter contained 99 patients with unknown pathologic outcomes. Patient characteristics of the excluded cohort of 176 patients can be found in Table S3. In the excluded cohort (n = 77) due to missing MRI or poor image quality, 26 (34%) were HR+/HER2−, 8 (10%) were HR+/HER2+, 7 (9%) were HR−/HER2+, and 36 (47%) were triple negative. There was no statistically significant difference in subtype distribution between the MRI-excluded cohort and the analysis cohort (*p* = 0.17). The pCR rate in the MRI-excluded cohort was 29% (22/77), slightly lower than the pCR rate in the analysis cohort (36%); however, *p*-value (*p* = 0.26) did not reach statistical significance.

### 3.2. Probability Threshold Analysis

**Figure 2:**
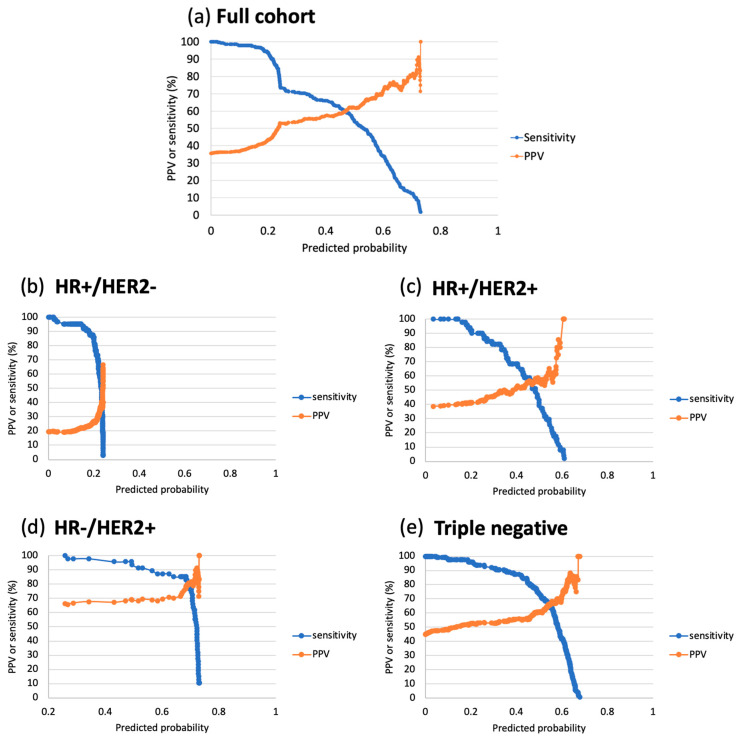
Plots of positive predictive value (PPV) and sensitivity versus predicted probability threshold. This plot was generated using the predictive model built in the full cohort and in hormone receptor (HR) and human epidermal growth factor receptor 2 (HER2) sub-cohorts: (**a**) Predicted probability was in the range of [0.00, 0.73], with corresponding ranges of [36%, 100%] and [100%, 2%] for PPV and sensitivity, respectively. (**b**) HR+/HER2−. The predicted probability was in the range of [0.00, 0.24], with corresponding ranges of [19%, 67%] and [100%, 3%] for PPV and sensitivity, respectively. (**c**) HR+/HER2+. The predicted probability was in the range of [0.03, 0.61], with corresponding ranges of [39%, 100%] and [100%, 2%] for PPV and sensitivity, respectively. (**d**) HR−/HER2+. The predicted probability was in the range of [0.03, 0.61], with corresponding ranges of [66%, 100%] and [100%, 11%] for PPV and sensitivity, respectively. (**e**) Triple negative. The predicted probability was in the range of [0.0002, 0.68], with corresponding ranges of [45%, 100%] and [100%, 1%] for PPV and sensitivity, respectively. In clinical application, a probability threshold was selected using a pre-specified PPV level, and the corresponding sensitivity can be used to evaluate the resulted PPV and sensitivity tradeoffs for the selected threshold.

When the probability threshold increased from minimum to maximum, the overall trend of increasing PPV and decreasing sensitivity was observed in the full cohort and all subtypes (Figure 2). However, PPV-sensitivity tradeoffs varied significantly among subtypes. In the HR+/HER2− sub-cohort, the maximum PPV was 67%. A steep increase in PPV, accompanied by a steep decrease in sensitivity, was observed when this subtype’s probability threshold was approaching the maximum value (0.24). In the HR−/HER2+ sub-cohort, however, PPV remained above 66% (the minimum) when the predicted probability threshold increased from the minimum to the maximum. A steep decrease in sensitivity was also observed when the probability threshold was approaching the maximum value (0.61) in this subtype.

Table 2 lists sensitivities when probability thresholds were set using minimum PPV levels of 50%, 70%, and 90%. With a PPV level of 50%, the model built in the full cohort achieved a sensitivity of 90%. However, sensitivity varied greatly among subtype-specific models. The lowest (20%, 95% CI: 10% to 30%) occurred in the HR+/HER2− model, and the highest (100%) occurred in the HR−/HER2+ model, where the minimum PPV was 66%. Table 3 lists clinical implications by subtype when probability thresholds were set at PPV 50%. When the PPV level increased to 70%, the sensitivity of the full-cohort model decreased to 29%. However, the sensitivity of the HR−/HER2+ model was still the highest (87%, 95% CI: 78% to 97%]), followed by 52% (95% CI: 43% to 61%) in the triple-negative model. When the PPV level was set to 90%, the full-cohort model resulted in a sensitivity of 10% (95% CI: 7% to 14%). While the sensitivity was low in other subtype-specific models, it was still the highest in the HR−/HER2+—45% (95% CI: 30% to 59%). These results suggested that MRI alone may not achieve reasonable sensitivities without sacrificing PPV. Combining MRI and core biopsy may achieve a 90% or higher PPV with a reasonable sensitivity across subtypes.

**Figure 3:**
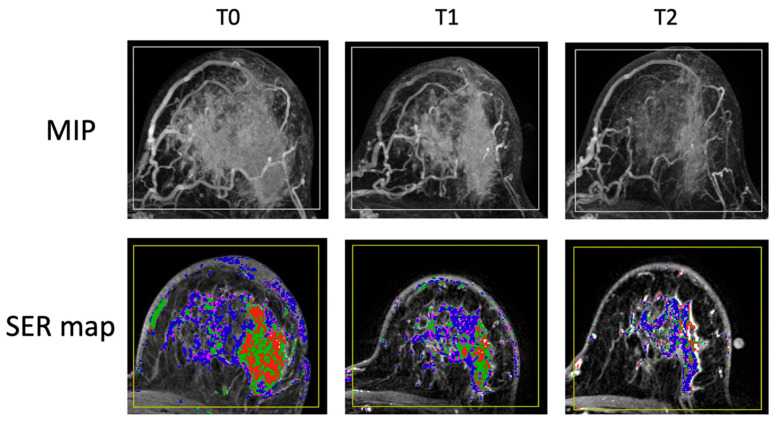
An image example of HR+/HER2− breast cancer. Columns represent treatment time points: T0—pretreatment, T1—early treatment, T2—inter-regimen. Top row: maximum intensity projection (MIP) of subtraction between early enhancement (approximately 2 min and 30 s post-contrast injection) and pre-contrast. Bottom row: signal enhancement ratio (SER) map overlaying on an axial MR image. Functional tumor volume is calculated as the sum of voxels above preset percentage enhancement (PE) and SER thresholds of 70% and 0 within the rectangular bounding box (yellow box). The FTVs are 150 cc (cubic centimeter) at T0, 75 cc at T1, and 44 cc at T2.

MR images of a representative case of HR+/HER2− breast cancer are shown in Figure 3. The patient started the treatment with a large FTV of 150 cc. At T2, the FTV reduced to 44 cc, resulting in a −70.7% change in FTV. This case represented a slow response to NAC, typically observed among HR+ breast tumors. According to the estimation of our subtype-specific model at T2, the probability of pCR was 0.18, lower than the threshold of PPV 50% for this subtype. Therefore, this case would be predicted as a non-pCR, and the patient would not be recommended to proceed to surgery at T2.

**Figure 4:**
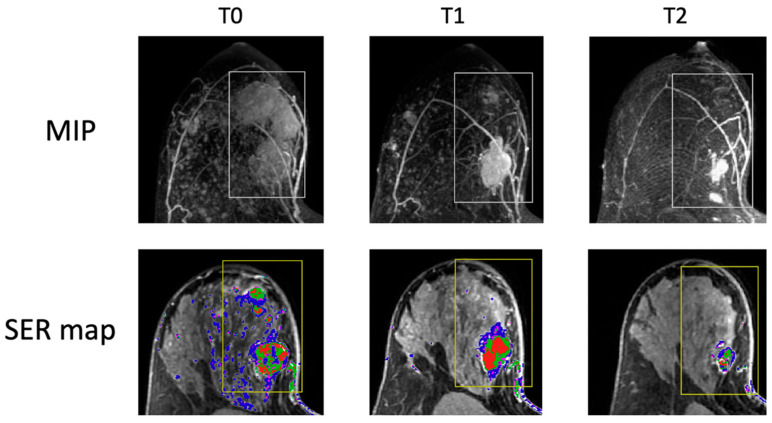
An image example of the HR−/HER2+ breast cancer. Columns represent treatment time points: T0—pretreatment, T1—early treatment, T2—inter-regimen. Top row: maximum intensity projection (MIP) of subtraction between early enhancement (approximately 2 min and 30 s post-contrast injection) and pre-contrast. Bottom row: signal enhancement ratio (SER) map overlaying on an axial MR image. Functional tumor volume is calculated as the sum of voxels above preset percentage enhancement (PE) and SER thresholds of 70% and 0 within the rectangular bounding box (yellow box). The FTVs are 38 cc (cubic centimeter) at T0, 13 cc at T1, and 3 cc at T2.

For comparison, MR images of a representative case of HR−/HER2+ breast cancer are shown in Figure 4. The patient started the treatment with a FTV of 38 cc. At T2, the FTV reduced to 3 cc, resulting in a −92.3% change in FTV. This case represented a fast response to NAC, typically observed among HER2+ breast tumors. According to the estimation of our subtype-specific model at T2, the probability of pCR was 0.70, higher than the thresholds set for PPV 50% and PPV 70% for this subtype. Therefore, this case would be predicted to have a pCR by MRI alone based on either a 50% PPV threshold or a 70% PPV threshold for HR+/HER2−. If this case were confirmed by core biopsy with no invasive disease at T2, the patient would be recommended to proceed to surgery early.

## 4. Discussion

In this study, we built FTV-based predictive models to predict pCR individually for each HR/HER2 subtype using data from the I-SPY 2 trial and observed different tradeoffs between PPV and sensitivity among HR/HER2 breast cancer subtypes when probability thresholds were selected based on predefined PPV levels. The presented predictive models and thresholds are now part of a treatment decision algorithm based on MRI and core biopsy histopathology to offer exceptional responders the option to skip anthracycline (AC) treatment in I-SPY 2 and go to surgery early. Patients who did not meet subtype-specific MRI thresholds or had residual according to histopathology evaluation would simply continue to AC treatment without interruption. The particular clinical application for treatment decision-making requires high PPV (correctly interrupt treatment) and reasonable sensitivity (correctly identify pCRs), while other MRI-based predictive models often report the tradeoff between sensitivity and specificity [17,18], offering an overall balanced view of prediction accuracy.

The results of this study are consistent with the known biology and treatment response characteristics of the HR/HER2-defined breast cancer subtypes. HR−/HER2+ and TN breast cancers tend to respond rapidly to treatment, with pCR rates up to 70% for HR−/HER2+ and in the range of 22–60% for TN in I-SPY 2 and other trials [19]. Conversely, HR+/HER2− breast cancers respond more slowly, achieving a low pCR rate in the range of 13–30%. Not surprisingly, in this subtype, the minimum PPV had to be relaxed to 50% to achieve a sensitivity of only 20%. By comparison, 100% sensitivity was achieved with a minimum PPV of 50% in the HER2+/HR− group.

This study utilized a large cohort, enabling sufficient sample sizes in HR/HER2 sub-cohorts. Models optimized for the four subtypes demonstrated different predictive performances assessed using AUC values. Interestingly, different FTV variables were selected in different subtype-specific models. Most models included FTV change at T2 as a predictor. However, the model for HR+/HER2+ only included FTV change at T1, i.e., further change in FTV did not lead to an additional increase in AUC. In addition, the model evaluated in the HR+/HER2+ sub-cohort also had the lowest AUC (0.68) compared to other subtypes (0.70 for HR+/HER2-, 0.73 for HR−/HER2+, and 0.74 for triple negative). This finding may reflect the heterogeneity of response among breast cancer subtypes and, particularly, in HR+/HER2+. It is well known that HER2+ tumors are more aggressive than HER2- tumors. However, HR status adds complexity to tumor response to NAC [20,21], which complicates the predictive performance of MRI as well. A single-institute study of 119 breast cancer patients observed a stronger association between radiological complete response (CR) for cancers with HR−/HER2+ status than those with HR+/HER2+ status, where radiological CR was assessed using ultrasound or MRI [22].

Breast cancer is genetically and clinically heterogeneous, and it is now a common practice to treat different breast cancer subtypes differently, using therapies that are more effective or preferentially suited for that subtype [23]. In the I-SPY 2 trial, HR, HER2, and MammaPrint status were used to assess drug efficacy, and patients were randomized to treatment arms based on their breast cancer subtypes [24]. DCE-MRI studies have also demonstrated differences in treatment response among breast cancer subtypes. In the I-SPY 1 trial, Hylton et al. reported that FTV performed differently in different breast cancer subtypes defined by HR and HER2 status in a multicenter study of 162 patients [25]. Despite these differences, FTV was predictive of recurrence-free survival in that study. Another retrospective study including 384 patients in I-SPY 2 reported a range of AUC values (between 0.67 and 0.74) among HR/HER2 subtypes when baseline FTV and percent change of FTV at T1, T2, and T3 (pre-surgery) were available for optimal model selection [14]. The West German Study Group (WSG) conducted the ADAPT (Adjuvant Dynamic Marker-Adjusted Personalized Therapy Trial Optimizing Risk Assessment and Therapy Response Prediction in Early Breast Cancer) trial, which focuses on improving risk assessment and predicting therapy response in early breast cancer by adjusting treatment based on dynamic markers [26]. A combined analysis of three sub-trials of WSG ADAPT reported different PPVs among HR+/HER2+, HR−/HER2+, and triple-negative subtypes using MRI after three weeks of NAC to predict pCR values of 0.42, 0.53, and 0.41, respectively in a smaller sample (n = 226) [27].

The probability threshold analysis of the current study demonstrated the potential clinical application of using MRI predictive models to identify good responders at the individual patient level. Low predicted probabilities of pCR were found for the HR+/HER2− subtype, which could be due to the majority of patients being non-pCRs. Thus, the model was indicating a low likelihood of achieving pCRs for this subtype. In clinical treatment decision-making, a subtype-specific probability threshold needs to be considered so that real responders with HR+/HER2− cancer will still have the option to proceed to surgery early. In this particular decision-making strategy, a high PPV (low false positive rate) was favored to minimally disrupt treatments while impacting patient care. However, PPV is affected by pCR rates, which explains why different PPV ranges were observed among different subtypes. For example, the overall PPV range was 19% to 67% in HR+/HER2−, where the pCR rate was the lowest (20%) among all subtypes. The PPV range was 66% to 100% in HR−/HER2+, where the pCR rate (66%) was the highest among all subtypes. Other studies using DCE-MRI to predict pCR also found variable PPVs among subtypes and low PPVs (21–28%) in HR+/HER2− [9,28,29]. Fukuda et al. reported a PPV of 21% (3/14) in the HR+/HER2− (Luminal) sub-cohort of 161 patients (pCR rate: 2%) [9]. Another observation we made was that the tradeoff between PPV and sensitivity varied among HR/HER2 subtypes. This observation can guide the probability threshold selection. Although higher PPV is desired for identifying good responders in all HR/HER2 subtypes, the probability threshold for the HR+/HER2− subtype needs to be set at a PPV level lower than 50% to avoid the potential steep drop in sensitivity (20% according to our results). At the same time, for HR−/HER2+, it is safe to set the PPV level at 70% for the threshold selection (with a sensitivity of 87%, according to our results).

Our study also indicated that FTV alone could not achieve a 90% or higher PPV while maintaining a reasonable sensitivity. Core biopsy of the tumor bed based on clip location is now performed at the 12-week inter-regimen timepoint for all I-SPY patients. To ensure that identified good responders are most likely pCRs (high PPV), a combined algorithm based on the predicted probabilities from MRI and core biopsy was used to make recommendations of proceeding to surgery early, provided that the thresholds are met for their tumor type and there is no invasive disease detected in pathology. In this algorithm, MRI thresholds will be trained and validated in combination with core biopsy to achieve a 90% or higher PPV with reasonable sensitivity. A pilot study involving a small cohort of 87 patients reported that the combined algorithm achieved a PPV of 92% (range: 83–100%) and a 53% sensitivity (range: 33–62%) when MRI thresholds were set at a 50% PPV level [30].

However, core biopsy is an invasive and painful procedure. In our future study, we will test the combination of MRI and liquid biopsy biomarkers such as circulating tumor DNA (ctDNA) or with genomic or proteomic biomarkers to further improve the performance of the combined model.

This study has several limitations. First, there is a potential mismatch between the retrospective data used to build MRI models and the prospective data on which the models will be applied. In the retrospective data, all patients completed two regimens (including AC) before their pathologic outcomes were assessed. However, in the prospective data, all patients with a predicted pCR will have the option to go to surgery and have their pathologic assessment without receiving AC. The actual performance of the presented MRI model will likely be lower in the prospective study. Other possible mismatches include different pCR rates due to variabilities in drug efficacies, different baseline FTV distribution in the subtype cohort, and lymph node status, which was not considered in the MRI model. The second limitation is the variability of sample sizes in HR/HER2 sub-cohorts due to the nature of clinical trial enrollment. In this study, HR−/HER2+ had the smallest sample size among all subtypes, which may cause overfitting in model optimization and limit the possibility of testing additional MRI biomarkers. The third limitation is that the variability in FTV calculation, caused by either uncertainty in protocol adherence [31], FTV segmentation [32], or treatment regimen, may affect the predictive performance of FTV. Lastly, because PPV is impacted by pCR prevalence, the proposed subtype-specific probability threshold selection method cannot achieve very high PPVs in subtypes with a low prevalence of pCR. Among these limitations, pCR rates, variability in FTV calculation, and treatment regimen may affect the generalizability of our findings. However, precautions were taken to limit the effect. Cross-validation was applied in the model development and probability threshold selection for the decision-making, with histopathology findings by core biopsy being trained and validated in separated cohorts.

In summary, this study presents the results of subtype-specific MRI models and probability threshold selection for identifying good responders in NAC. These findings lay the groundwork for integrating MRI with core biopsy or liquid biopsy and/or genomic data to test the performance of predicting pCR. The combined algorithm is now integrated into the prospective design of I-SPY2, and hundreds of patients have been tested prospectively using the algorithm [33]. In our future work, we will perform prospectively validation of the FTV-based subtype-specific models along with probability thresholds in a larger cohort of I-SPY 2.

## Figures and Tables

**Table 1:** Patient characteristics.

Characteristic	Eligible * n = 990	Analysis n = 814	*p*-Value
Age (in years, mean ± standard deviation)	48.8 ± 10.6	49.1 ± 10.5	0.51
HR/HER2 subtype (n, %)			0.81
HR+/HER2−	380 (38%)	328 (40%)	
HR+/HER2+	156 (16%)	132 (16%)	
HR−/HER2+	89 (9%)	71 (9%)	
HR−/HER2− (triple negative)	363 (37%)	283 (35%)	
Unknown	2 (0.2%)	0 (0%)	
Menopausal status (n, %)			0.904
Premenopausal	464 (47%)	373 (46%)	
Perimenopausal	33 (3%)	30 (4%)	
Postmenopausal	291 (29%)	253 (31%)	
Not applicable	134 (14%)	102 (13%)	
Unknown	68 (7%)	56 (7%)	
Pathological outcome (n, %)			NA **
pCR	311 (31%)	289 (36%)	
Non-pCR	580 (59%)	525 (64%)	
Unknown	99 (10%)	0 (0%)	

* Eligible for enrollment. ** The *p*-value of pCR distribution was not calculated because a large number of patients in the eligible cohort were missing pathological outcomes. HR: hormone receptor. HER2: human epidermal growth factor receptor 2. pCR: pathologic complete response. *p*-values were calculated using a two-sided t-test for age and Fisher’s exact test for HR/HER2 and menopausal status.

**Table 2:** Sensitivities of predicting pathologic complete response at inter-regimen using probability threshold based on the positive predictive value (PPV) level.

Cohort	PPV Level 50		PPV Level 70		PPV Level 90	
	Probability Threshold	Sensitivity (95% CI) (%)	Probability Threshold	Sensitivity (95% CI) (%)	Probability Threshold	Sensitivity (95% CI) (%)
Full	0.23	90 (87, 94)	0.59	29 (24, 35)	0.72	10 (7, 14)
HR+/HER2−	0.24	20 (10, 30)	+Inf	0	+Inf	0
HR+/HER2+	0.34	78 (67, 90)	0.57	16 (6, 26)	0.60	8 (0.5, 15)
HR−/HER2+	0	100	0.62	87 (78, 97)	0.72	45 (30, 59)
Triple negative	0.13	98 (95, 100)	0.58	52 (43, 61)	0.67	4 (0.6, 7)

HR: hormone receptor. HER2: human epidermal growth factor receptor 2. +Inf: the set PPV level was higher than the maximum predicted probability in the cohort, resulting in no patients being predicted to have a pCR.

**Table 3:** Clinical implications by breast cancer subtype using the MRI model.

Subtype	Sensitivity at PPV 50%	Clinical Implication of Recommendation
HR+/HER2−	20%	20% of true pCR cases proceed to surgery early, avoiding unnecessary therapy. 80% of true pCR cases continue to NAC, as planned.
HR+/HER2+	78%	78% of true pCR cases proceed to surgery early, avoiding unnecessary therapy. 22% of true pCR cases continue to NAC, as planned.
HR−/HER2+	100%	100% of true pCR cases proceed to surgery early, avoiding unnecessary therapy. 0% continue to NAC, as planned.
Triple negative	98%	98% of true pCR cases proceed to surgery early, avoiding unnecessary therapy. 2% of true pCR cases continue to NAC, as planned.

HR: hormone receptor. HER2: human epidermal growth factor receptor 2. NAC: neoadjuvant chemotherapy. pCR: pathologic complete response.

## Data Availability

The data presented in this study are openly available in the Cancer Imaging Archive at 10.7937/TCIA.D8Z0-9T85 and 10.7937/tcia.kk02-6d95.

## Supplementary Materials

The following supporting information can be downloaded at: https://www.mdpi.com/article/10.3390/tomography11030026/s1, Figure S1: An example of FTV measurement. FTV is calculated within the manually delineated volume of interest (VOI) by summing up voxels above the percent enhancement (PE) and signal enhancement ratio (SER) thresholds; Figure S2: Comparison of models in individual sub-cohorts at inter-regimen; Table S1: Standardized I-SPY 2 DCE-MRI acquisition parameters; Table S2: Model determination and evaluation for the full cohort and for individual subtype cohorts; Table S3 Patient characteristics of the excluded cohort.
