# Supplementary material for: MRI-Based Model for Personalizing Neoadjuvant Treatment in Breast Cancer
Source: Tomography. 2025 Feb 27;11(3):26. doi: 10.3390/tomography11030026 (PMC11946387; doi:10.3390/tomography11030026)
Supplement: Supplementary file 1 [file tomography-11-00026-s001.zip › tomography-3353579-supplementary.pdf]

Supplemental Materials

Table S1 Standardized I-SPY 2 DCE-MRI acquisition parameters

Parameter	Value
Sequence type	Gradient echo (GE)
2D or 3D sequence	3D
Slice orientation	Axial
Laterality	Bilateral
Frequency direction	A/P
Phase direction	R/L
FOV - frequency	260 – 360 mm
FOV - phase	260 – 360 mm
Matrix – frequency (acquired)	384 – 512
In-plane resolution	≤ 1.4 mm
Fat-suppression	Active fat-sat recommended
TR	4 – 10 ms
TE	Minimum TE
Flit angle	10 – 20 degrees
Slice thickness (acquired, not interpolated)	≤ 2.5 mm
Number of slices	≥ 60; complete bilateral coverage
Slice Gap	No gap
Parallel imaging factor	≤ 2
No. of excitations or averages	≤ 2
k-space ordering	-k to +k (standard, non-centric)
Sequence acquisition time	80 sec ≤ scan time ≤ 100 sec
Total post-contrast imaging duration	≥ 8 minutes following injection

Table S2 Model determination and evaluation for the full cohort and for individual subtype cohorts. Predictive models employing logistic regression were developed for the full cohort and individual HR/HER2 subtypes to predict pCR (versus non-pCR) utilizing FTV variables available up to T2. The model built in the full cohort had HR/HER2 subtype information as a covariate in addition to the FTV variables because HR/HER2 subtype is a known predictor of pCR [25,26]. The area under the receiver operating characteristic (ROC) curve (AUC) and 95% confidence intervals (CIs) were used to estimate the predictive performance of all models. For models with multiple predictors, AUCs were calculated using 10-fold cross-validation, and 95% CIs were estimated using 1,000 bootstrap resamples. Differences in AUCs were tested using a bootstrapping method with 2,000 replicates. The final version of the model was established by comparing point estimates of AUC values of models with all combinations of FTV variables. The model with highest AUC was assigned as subtype-specific model for individual HR/HER2 subtypes. Non subtype-specific models, used for comparison with subtype-specific models, simply consisted of all FTV variables, i.e. baseline FTV, change of FTV at T1, and change of FTV at T2, for all breast cancer subtypes.

Cohort	N (pCR rate)	Model	Included FTV variables	AUC (95% CI)
Full*	814 (36%. 289/814)		FTV0, %ΔFTV0_1, %ΔFTV0_2	0.76 (0.73, 0.80)
HR+/HER2-	328 (20%, 64/328)	Subtype-specific	%ΔFTV0_2	0.70 (0.63, 0.78)
		Non subtype-specific	FTV0, %ΔFTV0_1, %ΔFTV0_2	0.65 (0.55, 0.76)
HR+/HER2+	132 (39%, 51/132)	Subtype-specific	%ΔFTV0_1	0.68 (0.59, 0.77)
		Non subtype-specific	FTV0, %ΔFTV0_1, %ΔFTV0_2	0.66 (0.57, 0.78)
HR-/HER2+	71 (66%, 47/71)	Subtype-specific	%ΔFTV0_2	0.73 (0.61, 0.86)
		Non subtype-specific	FTV0, %ΔFTV0_1, %ΔFTV0_2	0.69 (0.53, 0.85)
Triple negative	283 (45%, 127/283)	Subtype-specific	FTV0, %ΔFTV0_2	0.74 (0.69, 0.80)
		Non subtype-specific	FTV0, %ΔFTV0_1, %ΔFTV0_2	0.73 (0.53, 0.85)

N: number of patients. HR: hormone receptor. HER2: human epidermal growth factor receptor 2. pCR: pathologic complete response. FTV: functional tumor volume. FTV0: baseline FTV. %ΔFTV0_1: percent change of FTV at early treatment. %ΔFTV0_2: percent change of FTV at inter-regimen. AUC: area under the receiver operating characteristic curve 95% confidence interval. FTV variables: lists of FTV variables included in the model. Subtype-specific: model was optimized and fit within the subtype cohort. Non-subtype-specific: model was not optimized and all FTV variables — FTV0, %ΔFTV0_1, %ΔFTV0_2, were included in the model.

Table S3 Patient characteristics of the excluded cohort

characteristic	Excluded cohort n=176
Age (in years, mean ± standard deviation)	47.1 ± 10.8
HR/HER2 subtype (n, %)	
HR+/HER2-	52 (30%)
HR+/HER2+	24 (14%)
HR-/HER2+	18 (10%)
HR-/HER2- (triple negative)	80 (45%)
Unknown	2 (1%)
Menopausal status (n, %)	
Premenopausal	91 (52%)
Perimenopausal	3 (2%)
Postmenopausal	38 (22%)
Not applicable	32 (18%)
Unknown	12 (7%)
Pathological outcome (n, %)	
pCR	22 (13%)
Non-pCR	55 (31%)
Unknown	99 (56%)

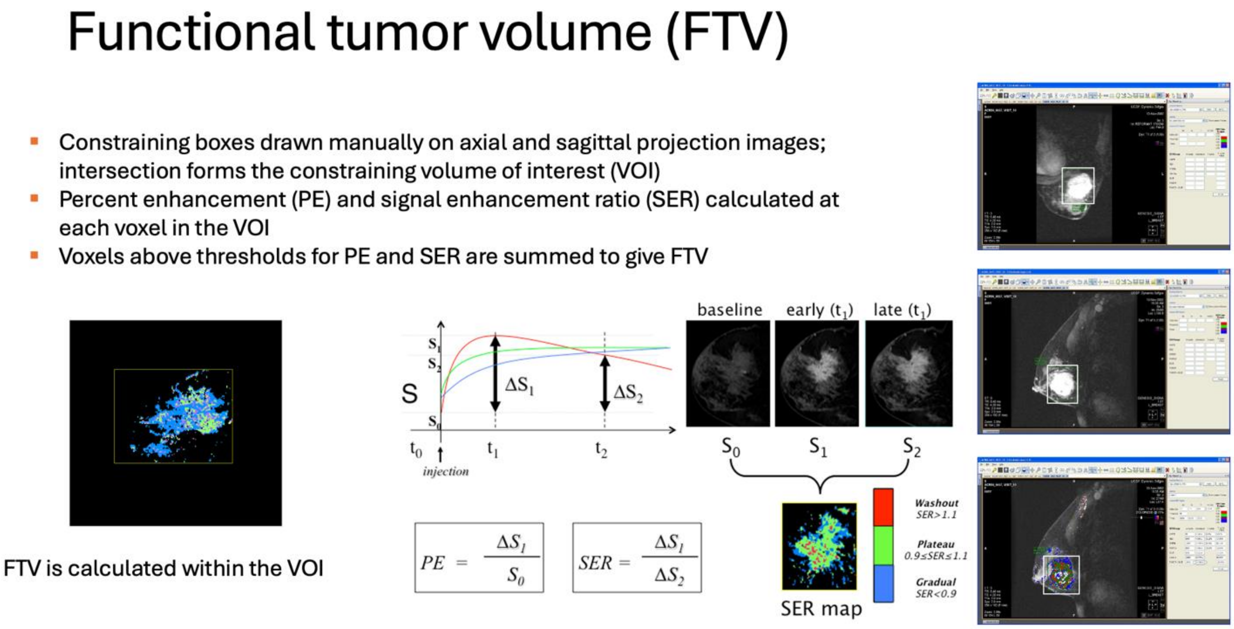

Figure S1 An example of FTV measurement. FTV is calculated within the manually delineated volume of interest (VOI) by summing up voxels above the percent enhancement (PE) and signal enhancement ratio (SER) thresholds.

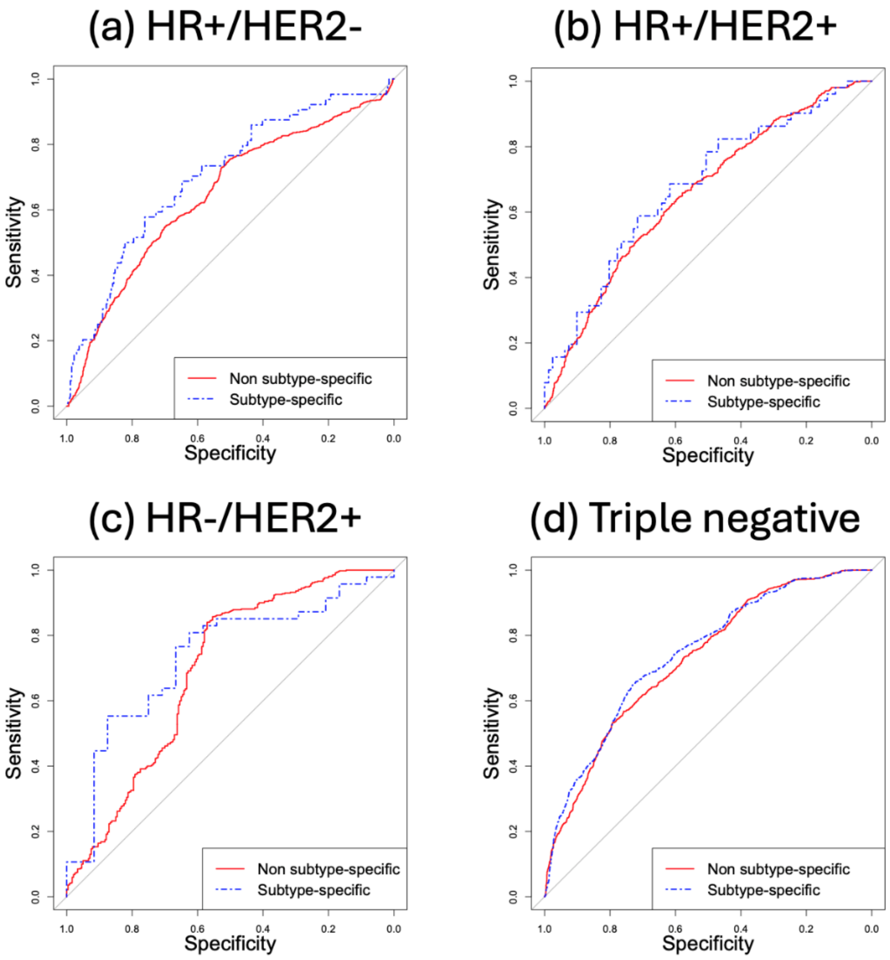

Figure S2 Comparison of models in individual sub-cohorts at inter-regimen. (a) HR+/HER2-; (b) HR+/HER2+; (c) HR-/HER2+; (d) triple negative. “Non subtype-specific” model refers to the logistic model with both baseline functional tumor volume (FTV) and percent change of FTV. “Subtype-specific” model refers to the optimal model generated by model optimization in the specific subtype cohort. Both ROC curves were estimated by “Non subtype-specific” and “Subtype-specific” models in the same cohorts. HR: hormone receptor. HER2: human epidermal growth factor receptor 2.
